# pDOCK: a new technique for rapid and accurate docking of peptide ligands to Major Histocompatibility Complexes

**DOI:** 10.1186/1745-7580-6-S1-S2

**Published:** 2010-09-27

**Authors:** Javed Mohammed Khan, Shoba Ranganathan

**Affiliations:** 1 Department of Chemistry and Biomolecular Sciences and ARC Center of Excellence in Bioinformatics, Macquarie University, NSW 2109, Australia; 2 Department of Biochemistry, Yong Loo Lin School of Medicine, National University of Singapore, 8 Medical Drive, Singapore 117597

## Abstract

**Background:**

Identification of antigenic peptide epitopes is an essential prerequisite in T cell-based molecular vaccine design. Computational (sequence-based and structure-based) methods are inexpensive and efficient compared to experimental approaches in screening numerous peptides against their cognate MHC alleles. In structure-based protocols, suited to alleles with limited epitope data, the first step is to identify high-binding peptides using docking techniques, which need improvement in speed and efficiency to be useful in large-scale screening studies. We present pDOCK: a new computational technique for rapid and accurate docking of flexible peptides to MHC receptors and primarily apply it on a non-redundant dataset of 186 pMHC (MHC-I and MHC-II) complexes with X-ray crystal structures.

**Results:**

We have compared our docked structures with experimental crystallographic structures for the immunologically relevant nonameric core of the bound peptide for MHC-I and MHC-II complexes. Primary testing for re-docking of peptides into their respective MHC grooves generated 159 out of 186 peptides with Cα RMSD of less than 1.00 Å, with a mean of 0.56 Å. Amongst the 25 peptides used for single and variant template docking, the Cα RMSD values were below 1.00 Å for 23 peptides. Compared to our earlier docking methodology, pDOCK shows upto 2.5 fold improvement in the accuracy and is ~60% faster. Results of validation against previously published studies represent a seven-fold increase in pDOCK accuracy.

**Conclusions:**

The limitations of our previous methodology have been addressed in the new docking protocol making it a rapid and accurate method to evaluate pMHC binding. pDOCK is a generic method and although benchmarks against experimental structures, it can be applied to alleles with no structural data using sequence information. Our outcomes establish the efficacy of our procedure to predict highly accurate peptide structures permitting conformational sampling of the peptide in MHC binding groove. Our results also support the applicability of pDOCK for *in silico* identification of promiscuous peptide epitopes that are relevant to higher proportions of human population with greater propensity to activate T cells making them key targets for the design of vaccines and immunotherapies.

## Background

The molecular machinery by which an antigen presenting cell (APC) presents T cell epitopes for recognition by T cell receptors (TR) and subsequent activation of T cells followed by the immune response cascade is fascinating. T cell epitopes are short antigenic peptide sequences (p) that are bound to and presented by the major histocompatibility complexes (MHC) for recognition by the TR [1]. These epitopes are essential subunit peptides that are required in order to stimulate cellular immune responses, especially the adaptive immune responses. Peptide epitopes can be of endogenous (processed within the cell) or exogenous (processed outside the cell) origins, which are presented for surveillance and recognition by the TR in an MHC allele and supertype dependant manner. Broadly classified into two types, MHC class I (MHC-I) complexes bind and present endogenous peptides whereas MHC class II (MHC-II) complexes prefer exogenous peptides. Typically, MHC-I proteins are heterodimers, consisting of a heavy α chain (I-ALPHA) of about 45 kDa, and a light chain, β2-microglobulin (Β2M) of about 12 kDa [2,3]. The α chain consists of α1 (G-ALPHA1), α2 (G-ALPHA2) and α3 (C-LIKE) domains where G-ALPHA1 and G-ALPHA2 domains form the peptide binding groove or ‘cleft’ [4]. MHC-II proteins are also heterodimeric proteins consisting of an α chain (II-APLHA; 34 kDa) and a β chain (II-BETA; 29 kDa) with very similar overall quaternary structure to that of MHC-I proteins [5-10]. However, their peptide binding groove is formed by the α1 and β1 domains of the two chains. 

Peptides presented by MHC-I are generally between 8-11 amino acids in length. These peptides are ‘chopped’ within the cytosol of the cell by cytosolic proteases and are transported to the MHC binding groove within the endoplasmic reticulum by the transporters associated with antigen processing (TAP) proteins in an ATP dependant manner. Following which, the peptides bind to the MHC to form the peptide-MHC (pMHC) complex which is then transported to the APC cell surface and presented for recognition by the TR of CD8^+^ cytotoxic T cells (CTLs). Similarly, the peptides presented by MHC-II are usually 12-25 amino acids in length and are endocytosed into the cell by the lysosomes where they bind the MHC-II proteins by displacing the original MHC-II ligand known as the ‘CLIP’ peptide to form the pMHC complex. And again, they are transported to the APC cell surface for recognition by the TR of the CD4^+^ T helper cells. Identification of true T cell epitopes from the repertoires of immunologically significant antigenic peptide sequences is a vital prerequisite in the process of conventional molecular vaccine design for prevention and treatment of infectious, autoimmune, allergic and graft *vs.* host diseases. The key step in TR-mediated immune response is thus the binding and presentation of antigenic endogenous or exogenous peptide epitopes, which can be reasonably well predicted using sequence-based methods for alleles with large datasets of known binding peptides, as reviewed earlier [11,12]. 

Experimental identification of T cell epitopes is a tedious, time consuming and expensive process owing to the large number and diversity of both MHC alleles and the antigenic peptides. Not to mention, is the extremely low chance of immunogenicity (1 in 2000 peptides) even amongst the peptides that bind strongly to the MHC (50%) [13]. Recently developed computational methods have proven to be vastly time and cost efficient in screening the vast oceans of peptides and MHC repertoires [14]. Current computational methods can be broadly classified into: 1. Sequence-based approaches which use sequence motifs [15], matrix models [16,17], Artificial Neural Network [18-20], Hidden Markov Model [21] and Support Vector Machine [22-24] for large-scale screening of potential T cell epitopes from protein sequence databanks and 2. Structure-based approaches such as protein threading [25,26], homology modeling [27,28], rigid docking [29] and flexible docking [2,3] which utilize three-dimensional data for detailed structural analysis of interactions between the MHC and bound segmental antigenic peptides. The former are more suitable for large-scale screening of potential T cell epitopes, while the latter work better for detailed analysis of short immunogenic regions of antigens [2]. Although sequence-based methods are well established, a major limitation of such techniques is the heavy reliance on the availability of large comprehensive training sets of peptides. Thus, these approaches are not appropriate for accurate prediction of peptides in circumstances where the data available is insufficient. Therefore, the coverage of sequence-based techniques is limited to subsets of binding peptides that belong to the most numerous groups and cannot generate reliable data for peptides that are least represented in the dataset [2], leaving structural immunoinformatics as the only option for such peptides [3,5-7]. 

Antigenic peptides that bind strongly to MHC alleles are known to elicit T cell responses [1-3,5-7,11]. Hence, their identification is a vital first step in the process of structure-based immune epitope prediction. The usual approach adopted to address this important issue is to utilize a powerful concept, based on the principle of structure-based drug design called “docking”, where peptides are computationally placed in MHC grooves in the best orientation,  reflecting steric and electrostatic complimentarity, using structure-based docking techniques. The accuracy with which the peptides are docked is measured in terms of Root Mean Square Deviation (RMSD) values obtained by comparing the docked conformations of the peptides to their original bound conformations in the respective X-ray crystal structures. With the development of new structural modeling and docking techniques and an increase in the number of protein structures deposited in the Protein Data Bank (PDB) [30] and the IMGT/3Dstructure-DB [31,32], structure-based approaches are being more commonly used to predict potential T cell epitopes [33], often producing modeled structures accurate to within 2.00Å RMSD from the experimental crystal structure, providing a wealth of information for structural analysis and the development of prediction methods. 

The development of an accurate protocol for flexible docking has helped us to successfully carry out quantitative predictions for both MHC-I and MHC-II alleles even with limited binding peptide data [3,5-7]. Our earlier docking protocol consisted of three steps (extended to four for pMHC-II complexes for incorporating the flanking residues on either side of the nonameric core, which is the 9-mer anchored to the MHC molecule): (1) rigid docking of the peptide nonamer termini into the MHC binding groove; (2) loop closure of central residues by satisfaction of spatial constraints; (3) followed by iterative ab initio refinements of ligand backbone and; (4) extension of flanking peptide residues by satisfaction of spatial constraints [2,3] (only for MHC-II related peptides). While accurate, this approach has multiple steps, resulting in suboptimal computational speeds. Therefore, the efficiency of this protocol for peptide docking to MHC needs to be improved for large-scale screening of T cell epitopes. A grid-based docking methodology has earlier been reported [34] to be highly accurate in pMHC docking over a limited MHC-I data. Hence, we have developed a grid-based peptide docking method (pDOCK) and have extensively tested it on both MHC-I and MHC-II peptides. The motivation behind the development of a faster and more accurate peptide docking methodology was to eventually improve the qualitative and quantitative efficacy of structure-based T cell epitope prediction. 

**Figure 1:**
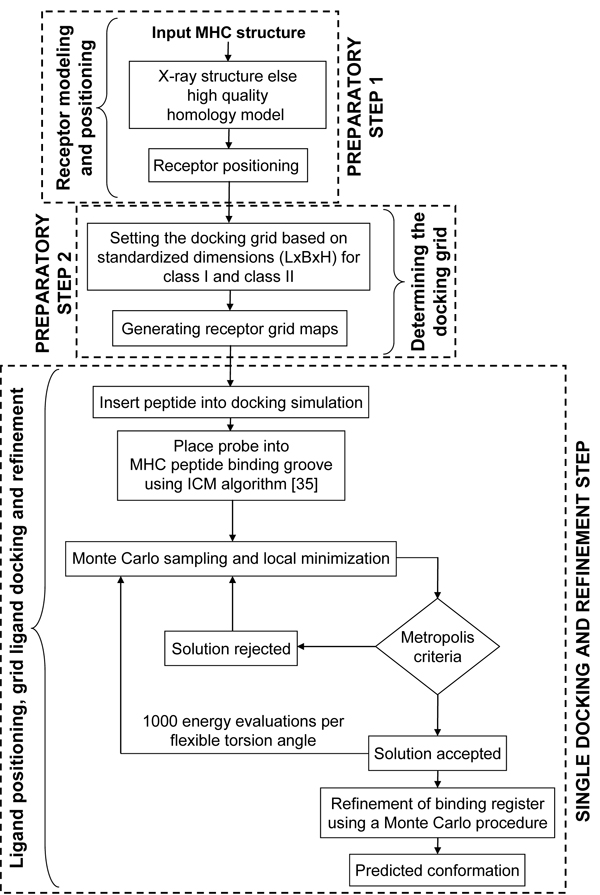
**Flowchart of the pDOCK protocol used in this work.** The two preparatory steps followed by a single consolidated docking and refinement step, are shown here.

In this study, we present pDOCK: a new computational technique for rapid and accurate docking of flexible peptides to the MHC receptors and primarily apply it to re-dock a non-redundant dataset of 186 (149 MHC-I and 37 MHC-II related) peptides, from MPID-T2 (http://biolinfo.org/mpid-t2) database for which X-ray crystal structures are available in the PDB and the IMGT/3Dstructure-DB, back into their respective MHC grooves. pDOCK comprises of two input preparatory steps followed by a single consolidated docking and refinement step as depicted in Figure 1. The pDOCK protocol involves: Preparatory step 1: receptor modeling and positioning; Preparatory step 2: determining the docking grid by defining the grid dimensions (length x breadth x height) for ligand placement and grid map generation within the vicinity of the receptor’s binding site and; Final docking and refinement step: ligand positioning within the grid, flexible docking of the peptide into the peptide binding groove and refinement of all ligand and binding site residues using the Internal Coordinate Mechanics (ICM) global optimization, docking algorithm [35] and a biased Monte Carlo procedure (see Methods section for more details). Our preliminary analysis of all pMHC complexes from the MPID-T2 database has provided us with standardized dimensions for the 3-D docking grids for both class I and class II pMHC structures. These standardized values were used to set the dimensions of the docking grids in all our experiments. Unlike the previously reported grid-based docking method [34], homology model building for MHC receptors has not been used in the development of pDOCK, instead using only experimentally determined X-ray crystallographic structures. The pDOCK method, however, is generic and is applicable to high quality homology models of alleles when experimental structures are not available. Here, the receptor modeling sub-step mentioned in the preparatory step 1 (Figure 1) can be used in the absence of structural data for the MHC proteins. Thus, the direct use of X-ray crystal structures in our docking simulations ensures accurate results.   

The first experiment that we conducted was to ensure that an extended peptide bound to its cognate MHC receptor preferentially selecting the same nonameric core peptides as in the crystal structure and then to evaluate the accuracy of the docked peptide. Hence, we performed re-docking of 186 peptides back to their cognate MHC receptors to check for conformational accuracy of the predicted binding registers and their Cα RMSD against their respective crystal structures. We have then benchmarked pDOCK with our earlier docking protocol [2,3] for a dataset of 50 selected (35 MHC-I and 15 MHC-II) pMHC complexes to verify the speed and accuracy of pDOCK against our earlier method. This was followed by validation and accuracy checks for pDOCK against available flexible peptide docking results obtained from the literature for a dataset of 15 peptides.

In the process of selecting immunogenic peptides for vaccine design, the two main aspects are to determine: (1) multiple peptides that bind to the same allele or MHC molecule and; (2) promiscuous or same peptides that bind multiple alleles. Therefore, as a secondary experiment, we have pursued to test the efficacy and robustness of our docking protocol in modeling the bound conformations of novel peptides to specific MHC alleles by carrying out docking of multiple peptides to a single MHC template structure (same MHC allele), suitable for immune epitope prediction from an antigenic protein, using a moving window of 9-mers along the entire sequence [3,5-7]. Our third experiment was to dock a single peptide from particular PDB structures onto multiple MHC templates (multiple alleles) from other crystal structures, suitable for determining promiscuous peptides capable of binding to a set of related alleles and therefore, important for vaccine design.

The Cα RMSD values have been calculated only for the nonameric core of the peptide (for both MHC-I and MHC-II related peptides) which is a contiguous immunogenic segment that forms the “binding register” within the MHC peptide binding cleft, as reported earlier by our group [3]. For the peptides with nine and less number of amino acid residues the entire peptide was used for Cα RMSD calculation. pDOCK accurately detected all 186 binding registers, i.e., the nonameric cores of the peptides are identical to their respective crystal structures. pDOCK generated 85.5% of all the peptides with Cα RMSD of less than 1.00 Å compared to their respective X-ray crystal structures. Our benchmarking results imply up to 2.5 fold improvement in the accuracy of the new peptide docking methodology. The validation results represent a sevenfold improvement in the accuracy of our technique compared to that of the existing methodologies in flexible docking and modeling of peptides into MHC grooves. Amongst the 21 peptides docked in the second experiment, the Cα RMSD values for docked peptides compared to their respective crystal structures were below 1.00 Å for 20 peptides (details in Results and discussion section). The third experiment accounted for all 4 peptides docked with less than 1.00 Å Cα RMSD compared to the same peptides from the corresponding template crystal structures (details in Results and discussion section). Overall, pDOCK is up to 60% faster than our earlier protocol and hence provides a rapid and accurate docking method to evaluate pMHC binding for large scale immune-epitope prediction. 

## Results and discussion

The fact that our earlier method was comparatively slower and that it involved rigid-docking of the peptide termini, acted as the platform for us to ‘revisit’ our pMHC docking methodology. Based on these requirements, we have developed a single step pMHC docking protocol (details in Methods section) as shown in Figure 1, which allows flexibility over the entire length of the peptide antigen and can be used as a generic method to obtain the conformations of bound peptide ligands to MHC binding grooves of both class I and class II MHC proteins. A systematic evaluation of pDOCK is performed as three separate tests: (1) exhaustive re-docking of all non-redundant peptides to their respective MHC grooves as a test case, benchmarking and validation; we then address two very significant practical problems faced by immunologists during the process of allele-specific peptide vaccine design: (2) the docking of multiple peptides that bind to same MHC allele, for immunogenic epitope scanning of antigenic sequences and; (3) docking of promiscuous peptides or same peptides binding to multiple MHC alleles for vaccine design, based on groups of disease-implicated alleles. A correctly docked structure is defined as the peptide with at most 2.50 Å Cα RMSD from the respective experimental X-ray crystal structure [2]. pDOCK has also been benchmarked against our previous docking protocol and validated on published peptide modeling and docking results from the literature. Bordner and Abagyan [34] suggested that while grid-based docking could be applied for pMHC-II, it was a more difficult problem. pDOCK has been successfully applied for MHC-II peptide docking as well with excellent results. 

### Experiment 1

#### *Re-docking bound peptides to their cognate MHC grooves*

**Figure 2:**
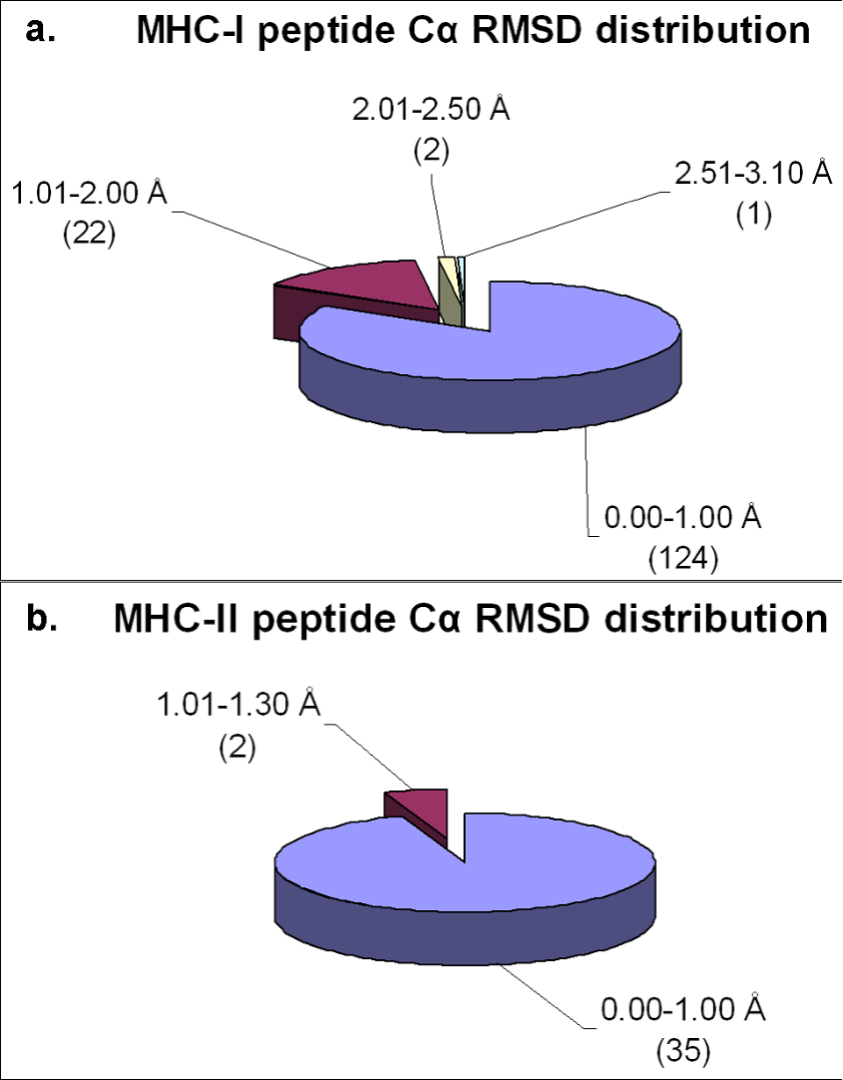
**Distribution of Cα RMSD of the docked peptides and their respective crystal structures across the non-redundant MPID-T2 dataset for peptides for a. MHC-I complexes and b. MHC-II complexes.** Most of the peptides from both MHC-I (124/149; 83%) and MHC-II (35/37; ~95%) datasets have their Cα RMSD values below 1.00 Å, highlighting the accuracy of our docking protocol. The number of peptides in each Cα RMSD range is given in parentheses.

pDOCK has been applied on a non-redundant dataset of 186 (149 MHC-I and 37 MHC-II) pMHC complexes from the MPID-T2 database (details in Methods section, data and docking results in Additional File 1 – Table S1). Initially, the peptides were extracted from the experimental pMHC complexes, randomized and set to extended conformations. This was followed by optimization of the peptide ligands and re-docking of the separated peptides back to their respective MHC grooves. As depicted in Figure 2, our technique generated 159 out of 186 peptides with Cα RMSD values less than 1.00 Å: 124 out of 149 peptides (83%) and 35 out of 37 peptides (~95%) for class I and class II MHC proteins, respectively. ~15% (22/149) and ~1% (2/149) of the peptides have their Cα RMSD values within the ranges 1.01-2.00 Å and 2.01-2.50 Å, respectively amongst the MHC-I peptides docked (Figure 2a). Similarly, ~5% of the peptides have their Cα RMSD values within a range of 1.01-1.30 Å amongst the MHC-II related peptides that were docked using pDOCK (Figure 2b). On an average, pDOCK resulted in a Cα RMSD value of about 0.56 Å for re-docking of peptides into their respective MHC grooves over the entire dataset of 186 pMHC complexes. 

**Figure 3:**
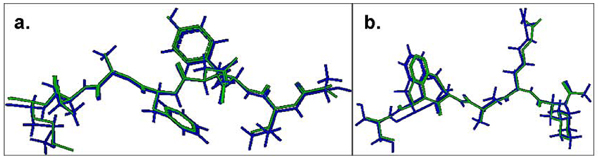
**Comparison of the lowest energy predicted and the experimental structures of the cognate peptides with the least RMSD values across the pDOCK test set. a. KAVYNFATM peptide in the MHC-I complex 1s7q (PDB code). b. XXRXXX peptide in the MHC-II complex 1d5x (PDB code).**  The peptides are shown in stick representation of all heavy atoms. The Cα RMSD values between the lowest energy docked conformation (green) and the native conformation of the bound peptides (blue) for the MHC-I structure 1s7q (PDB code) and the MHC-II structure 1d5x (PDB code) are 0.09 Å and 0.11 Å, respectively. X: Amino acid analogues (chemical mimics).

Our best results are shown in Figure 3, with structural comparison between the lowest energy docked conformation and the native conformation of the bound peptides for MHC-I (PDB code 1s7q) and MHC-II (PDB code 1d5x) structures. These docked conformations of peptide structures have the best Cα RMSD values of 0.09 Å and 0.11 Å respectively, obtained over the entire dataset. The MHC-II peptide in Figure 3b has 5 out of 6 amino acid residues replaced by amino acid analogues (chemical mimics) in the crystal structure. Nonetheless, it has the best Cα RMSD value among all the MHC-II related peptides used in this study, supporting pDOCK’s applicability to peptide or peptide analogues (containing amino acid mimics in structure-based drug design). pDOCK also generated the least energy docked orientations for all the peptides with accurate determination of their respective binding registers, i.e. having the exact nonameric core in the binding grooves, with respect to their native bound conformations in the X-ray crystal structures. All peptides except one from the class I pMHC crystal structure (PDB code 2gtw; Cα RMSD of 3.08 Å) were within the acceptable 2.50 Å Cα RMSD from their respective native conformations (Figure 2a). Also, none of the MHC-II related peptides showed any deviation from the acceptable 2.50 Å Cα RMSD threshold (Figure 2b). 

**Figure 4:**
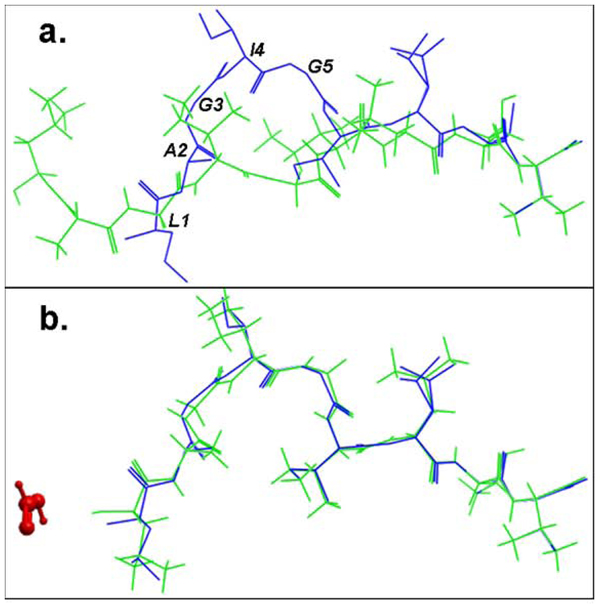
**Structural comparison of the lowest energy docked conformations and the experimental structures of the bound peptide for the pMHC structure 2gtw when the formic acid molecule was a. omitted and b. included in the docking simulation.** The peptides are shown in stick representation of all heavy atoms. The Cα RMSD values between the lowest energy predicted (green) and the native conformation of the cognate peptide (blue) for the structure 2gtw when the formic acid molecule was omitted and included in the docking protocol were 3.08 Å and 0.24 Å respectively. The peptide residues of its native conformation that were not accurately docked in the absence of the formic acid molecule are labeled in black in (a). The formic acid molecule is depicted in red in (b).

We carefully examined the re-docked conformation of the peptide LAGIGILTV in the MHC groove of the complex 2gtw, with the X-ray structure. In 2gtw, peptide residues 1 to 5 interact with a formic acid molecule, which was not explicitly introduced into the docking simulation. When the formic acid molecule was included in the docking simulation, the predicted orientation of the peptide using pDOCK is energetically more favourable for pMHC complex formation than the predicted conformation when the formic acid molecule is omitted. The improvement in accuracy by the inclusion of the formic acid molecule is ~13 folds. This is portrayed in Figure 4 which clearly indicates that the peptide residues Leu 1, Ala 2, Gly 3, Ile 4 and Gly 5 that are not correctly predicted in the absence of the formic acid molecule (Figure 4a), are accurately docked when the formic acid molecule is introduced into the docking simulation (Figure 4b), resulting in an improvement in the Cα RMSD value from 3.08 Å to 0.24 Å.

Although water molecules and other common biological ions such as phosphate and chloride may mediate pMHC interactions in some cases, they were omitted from our experiments because the significance and contributions of these molecules towards pMHC binding vary immensely between different peptides and specific alleles over a large dataset like the one used in this study (186 complexes). Our previous protocol achieves a Cα RMSD of 1.53 Å for the bound structure of the peptide from pMHC complex 1jf1, due to the presence of a water molecule positioned around the peptide residues 5 to 7 in the crystal structure leading to erroneous prediction of the loop formed, which resulted in incorrect positioning of interacting residues [2]. However, pDOCK successfully overcomes this restriction to accurately predict the least energy bound conformation of this peptide with a Cα RMSD value of 0.30 Å. The enhancement in accuracy of docking is a direct consequence of the improved sampling of available conformational space in pDOCK. This preliminary experiment is a critical first step as it establishes the validity of our approach and helps us test the ability of our technique to accurately dock cognate peptides into their respective MHC receptors, using the proposed single-step docking procedure.

#### *Benchmarking with our previous methodology*

In order to ascertain the improvement in speed and accuracy of pDOCK compared to the old technique, we have benchmarked our peptide docking methodology with our earlier pMHC docking protocol [2,3] over a subset of 50 pMHC complexes (35 MHC-I and 15 MHC-II) from the complete non-redundant dataset (listed in Additional File 1 – Table S1) and the results are presented in Table 1. pDOCK results are consistently better than our earlier docking methodology in terms of accuracy (Cα RMSD) of the modelled or docked peptide compared to their X-ray crystal structures after docking back into their respective MHC grooves. The new protocol also generates the least energy docked conformations for all 50 peptides with Cα RMSD values less than 1.00 Å, compared to eight peptides, docked using the earlier method, having Cα RMSD values above 1.00 Å (graphically shown in Additional File 2 – Figure S1). The new procedure outmatches the old protocol particularly well for complexes 1s9y, 1hhh, 1jf1, 1e27, 1jpf, 1qo3, 1wbz and 1g7p (Table 1) amongst the MHC-I structures and for structures 1uvq and 1aqd (Table 1) amongst the MHC-II structures (highlighted in yellow in Additional File 2 – Figure S1). 

**Table 1:** Benchmarking pDOCK with our earlier methodology.

S. No.	Allele	PDB	Peptide Length	Peptide Sequence	Cα RMSD (Å)	
					
					Previous method	pDOCK
**MHC-I**						

1	HLA-A*1101	1qvo	10	Q**VPLRPMTYK**	0.53	0.24
2	HLA-A*0201	1qr1	9	IISAVVGIL	0.46	0.29
3	HLA-A*0201	1akj	9	ILKEPVHGV	0.87	0.39
4	HLA-A*0201	1i1y	9	YLKEPVHGV	0.70	0.66
5	HLA-A*0201	1i7r	9	FAPGFFPYL	0.59	0.47
6	HLA-A*0201	1i7u	9	ALWGFVPVL	0.32	0.29
7	HLA-A*0201	1oga	9	GILGFVFTL	0.32	0.16
8	HLA-A*0201	1qsf	9	LLFGYPVAV	0.54	0.34
9	HLA-A*0201	1lp9	9	ALWGFFPVL	0.58	0.26
10	HLA-A*0201	1s9y	9	SLLMWITQS	1.09	0.39
11	HLA-A*0201	1hhh	10	F**LPSDFFPSV**	1.10	0.49
12	HLA-A*0201	1jf1	10	E**LAGIGILTV**	1.53	0.30
13	HLA-B*0801	1agc	8	GGKKKYQL	0.28	0.23
14	HLA-B*0801	1mi5	9	FLRGRAYGL	0.42	0.37
15	HLA-B*2705	1ogt	9	RRKWRRWHL	0.51	0.18
16	HLA-B*2705	2a83	9	RRRWHRWRL	0.55	0.18
17	HLA-B*3501	2cik	9	KPIVVLHGY	0.74	0.26
18	HLA-B*3508	3bwa	8	FPTKDVAL	0.56	0.26
19	HLA-B*5101	1e27	9	LPPVVAKEI	1.27	0.18
20	HLA-B*5301	1a1m	9	TPYDINQML	0.59	0.28
21	HLA-Cw*0401	1im9	9	QYDDAVYKL	0.49	0.34
22	HLA-G*0101	2dyp	9	RIIPRHLQL	0.43	0.16
23	H2-Db	1fg2	9	KAVYNFATC	0.25	0.19
24	H2-Db	3buy	9	LSLRNPILV	0.63	0.23
25	H2-Db	1yn7	10	S**SLENFAAYV**	0.62	0.14
26	H2-Db	1jpf	11	SG**VENPGGYCL**	1.14	0.36
27	H2-Dd	1qo3	10	R**GPGRAFVTI**	1.49	0.17
28	H2-Kb	1t0m	8	SSIEFARL	0.66	0.21
29	H2-Kb	1vac	8	SIINFEKL	0.32	0.22
30	H2-Kb	1wbz	9	SSYRRPVGI	0.89	0.19
31	H2-Kb	1s7q	9	KAVYNFATM	0.20	0.09
32	H2-Kb	1g7p	9	SRDHSRTPM	0.97	0.17
33	H2-Kd	1vgk	9	SYVNTNMGL	0.86	0.25
34	H2-Kk	1zt1	8	FEANGNLI	0.57	0.45
35	H2-Ld	2e7l	9	QLSPFPFDL	0.37	0.35

**MHC-II**						

36	HLA-DQB1*0602	1uvq	20	MN**LPSTKVSWA**AVGGGGSLV	1.09	0.23
37	HLA-DRB1*0301	1a6a	15	PVSK**MRMATPLLM**QA	0.38	0.30
38	HLA-DRB1*0101	1aqd	14	GSD**WRFLRGYHQ**YA	1.08	0.28
39	HLA-DRB1*0101	1fyt	13	PK**YVKQNTLKL**AT	0.68	0.23
40	HLA-DRB1*0101	2iam	15	GEL**IGILNAAKV**PAD	0.56	0.24
41	HLA-DRB1*0401	1d5x	6	XXRXXX	0.23	0.11
42	HLA-DRB1*0401	1d5z	7	XXRAXSX	0.33	0.22
43	HLA-DRB1*0401	1d6e	8	XXRXMASX	0.32	0.14
44	HLA-DRB1*0401	1j8h	13	PK**YVKQNTLKL**AT	0.59	0.20
45	HLA-DRB3*0101	2q6w	11	A**WRSDEALPL**G	0.54	0.30
46	HLA-DRB5*0101	1fv1	20	NPVVHF**FKNIVTPRT**PPPSQ	0.88	0.59
47	I-Ad	1iao	14	RGI**SQAVHAAHA**EI	0.81	0.27
48	I-Ak	1iak	13	ST**DYGILQINS**RW	0.42	0.23
49	I-Au	2pxy	11	R**GGASQYRPS**Q	0.78	0.28
50	I-Ek	1r5v	13	ADLI**AYPKAATK**F	0.82	0.28

Cα RMSD values are calculated only for the nonamer binding cores (shown in bold) for peptides with more than 9 residues in the X-ray crystal structures. X: Amino acid analogues (chemical mimics). A graphical representation of the results is available in Additional File 2 – Figure S1.

These results suggest that some of the conformational limitations of our previous methodology, such as the presence of water molecules in and around the peptide and within the peptide binding groove in the original PDB structure, have been addressed in our new docking protocol making it highly accurate. Besides an improvement in the accuracy, pDOCK is also able to accurately model docked conformations for some peptides especially for MHC-II related peptides with more than 9 amino acid residues, thereby improving the coverage over the entire length of the peptides. Peptides from the pMHC complexes 1uvq and 2iam were among the highest coverage (20 and 15 residues respectively) obtained in this experiment with Cα RMSD values 0.42 Å and 0.46 Å respectively over the length of the entire peptide (results not shown). The reliability for the accurate prediction of flanking residues (especially for MHC-II peptides) depends upon their interactions with the MHC residues outside the peptide binding groove and therefore, have not been included in the calculation of Cα RMSD values reported. 

In terms of the computational time to complete a single docking experiment, pDOCK is up to 60% faster (on an average) than the earlier method as summarized in Table 2. The average time taken by pDOCK is approximately 10 min. (the preparatory receptor positioning step of ~3 sec. {0.50%}, determining the docking grid taking ~42.6 sec. {7.10%} and the single docking and refinement step of ~9.24 min. {92.4%}), compared to 23.50 to 24.50 min (Step 1 taking ~5 min., Step 2 of ~30 sec., Step 3 taking ~18 min. and Step 4, which was only applicable to MHC-II related peptides, of ~1 min.) using the old protocol on a 2 CPU 3.20 GHz 3 GB RAM workstation. The average time taken for each of the steps using either of the methodologies is calculated over the entire non-redundant dataset of 186 pMHC complexes catalogued in additional file 1 – table S1. The mean Cα RMSD value for the least energy docked conformations of peptides, from the dataset of 50 peptides used for benchmarking, was 0.27 Å using pDOCK compared to 0.65 Å for the old procedure. This denotes almost two and a half fold improvement in the accuracy of our novel docking strategy over a larger dataset (50 peptides) than that used previously (40 peptides) [2].       

**Table 2:** Comparison of computational time of pDOCK with our earlier docking method

Previous method	pDOCK
Step 1: ~ 5 *min*	
Step 2: ~ 30 *s*	Preparatory Step 1: ~ 3*s
Step 3: ~ 18 *min*	Preparatory Step 2: ~ 42.6 *s*
Step 4^#^: ~ 1 *min*	Single docking and refinement step: ~ 9.24 *min*

**Total: ~ 23.50 – 24.50 *min***	**Total: ~ 10 *min***

Both methodologies were applied using a 2 CPU 3.20 GHz 3 GB RAM workstation. *Only for X-ray crystal structures of MHC proteins. The time taken for this step would increase if homology modeling needs to be carried out. ^#^Applicable only to MHC-II related peptides.

#### *Validation against previously published studies*

Keeping in mind the essence of improving the accuracy and robustness of the proposed strategy, we have validated pDOCK with seven studies involving MHC-I peptide docking/modeling and one study involving MHC-II peptide docking, covering 15 pMHC structures and compared the results by re-running our earlier method. The results of our validation experiments are compiled into Table 3. Peptides 1, 2, 3, 4 and 15 (Table 3) are new in this study and are collated from recent publications [34,36,37], whereas the remaining 10 were from the validation studies reported for our earlier methodology [2]. To the best of our knowledge, these results represent a sevenfold increase in the accuracy of pDOCK compared to available flexible docking techniques in the remodeling of pMHC complexes. Interestingly, the validation criteria for almost all of the previously published studies [34,36-40] involved either docking or remodeling of peptides back into their original crystal structure. Although the Cα RMSD values (0.29 Å and 0.30 Å, respectively) for peptides 2 and 3 (Table 3) were slightly higher, they are still comparable with the Cα RMSD values reported earlier (0.23 Å and 0.22 Å, respectively) [34]. Peptide 1 (Table 3) however, was generated with a better Cα RMSD (0.31 Å) compared to the Cα RMSD (0.76 Å) reported in the same earlier grid-based docking study [34]. The enhancement in the accuracy for peptide 1 could be a direct implication of more conformational sampling space in a flexible environment resulting from a relatively larger docking grid (35.36 Å x 35.52 Å x 35.79 Å) for MHC-I peptides and a lower temperature (300 K) used in pDOCK compared to the grid dimensions (34 Å x 34 Å x 25 Å) and temperature (700 K) used in the previous grid-based docking study [34]. Thus, pDOCK is not only comparable to but also surpasses the available techniques in flexible docking and remodeling of peptides with regards to the accuracy (Cα RMSD) with which it predicts the bound structure of a peptide to its respective MHC groove. By and large, our results illustrate the advantages of using grid-based flexible docking over conventional docking protocols.

**Table 3:** Comparison of pDOCK with published MHC–peptide modeling and flexible docking methods.

S.No	Technique	Peptide Sequence	MHC class	PDB	RMSD (Å)		
					
					Published	Previousmethod	pDOCK
1	Grid-based Flexible docking [34]	RGYVYQGL	I	1kpu^#^	0.76	0.59	0.31
2	Grid-based Flexible docking [34]	ALWGFVPVL	I	1i7u	0.23	0.32	0.29
3	Grid-based Flexible docking [34]	ELAGIGILTV	I	1jf1	0.22	1.53	0.30
4	Monte Carlo annealing [37]	LLFGYPVYV	I	1duz^#^	3.01	0.33	0.33
5	Simulated annealing [38]	FLPSDFFPSV	I	1hhh	1.59	1.10	0.48
6	Simulated annealing [38]	GILGFVFTL	I	1hhi^#^	0.46	0.32	0.16
7	Simulated annealing [38]	ILKEPVHGV	I	1hhj^#^	0.87	0.87	0.55
8	Simulated annealing [38]	LLFGYPVYV	I	1hhk^#^	0.78	0.33	0.33
9	Combinatorial buildup algorithm [39]	RGYVYQGL	I	2vaa^#^	0.56	0.32	0.22
10	Combinatorial buildup algorithm [40]	LLFGYPVYV	I	1hhk^#^	1.40	0.33	0.33
11	Combinatorial buildup algorithm [40]	ILKEPVHGV	I	1hhj^#^	1.30	0.87	0.55
12	Combinatorial buildup algorithm [40]	GILGFVFTL	I	1hhi^#^	1.60	0.32	0.16
13	Multiple copy algorithm [41]	FAPGNYPAL	I	2vab^#^	2.70	0.40	0.25
14	Multiple copy algorithm [42]	GILGFVFTL	I	1hhi^#^	1.40	0.32	0.16
15	GOLD/GLIDE Flexible docking [36]	XXRXMASX	II	1d6e	1.24/3.06	0.32	0.14

X: Amino acid analogues (chemical mimics). ^#^These structures are not listed in Additional File 1 - Table S1 due to redundancy in MPID-T2.

**Figure 5:**
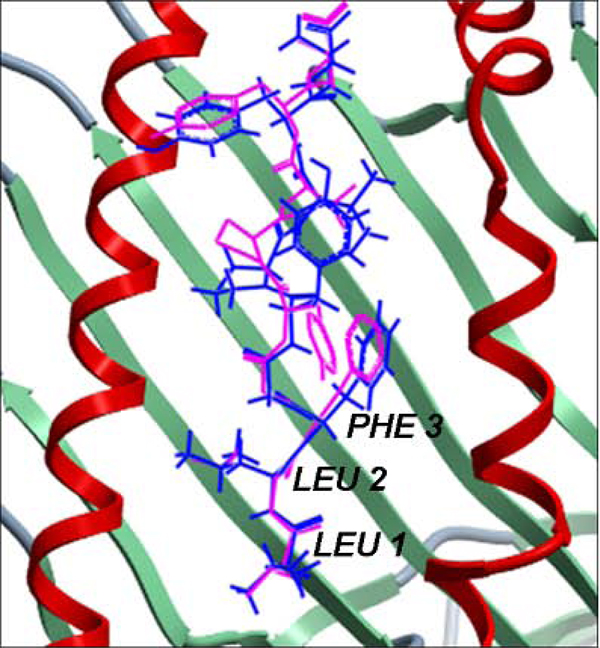
**Structural comparison between the native conformation and the lowest energy docked conformation of the cognate peptide in MHC-I complex 1duz.** The peptide is shown in stick representation wherein the native conformation is in pink and the docked conformation is in blue. The MHC peptide binding ‘groove’ is shown as ribbons. The Cα RMSD between the native and the lowest energy docked conformation of the bound peptide from our work is 0.33 Å which is up to three and a half times better than an earlier reported Cα RMSD of 3.01 Å [37]. The peptide residues of our lowest energy docked conformation that were better modeled and aligned to that of its native conformation when compared to the lowest energy docked conformation reported earlier [37] are labeled in black. This structure is not listed in Additional File 1 - Table S1 since it was a redundant structure in MPID-T2.

Figure 5 provides a pictorial representation of an example of the above discussed accuracy. This structural comparison between the least energy docked conformation generated using pDOCK and that of the native conformation of the cognate peptide in the complex 1duz portrays not only the highly accurate predicted conformation of the peptide, Cα RMSD of 0.33 Å compared to that of 3.01 Å reported earlier [37], but also highlights the fact that the peptide’s N-terminal residues (Leu 1, Leu 2 and Phe 3) were better modeled and structurally well aligned to that of its native conformation when compared to the lowest energy docked conformation reported earlier [37]. Notably, the least energy docked conformations generated for a common murine MHC (H2-Kb) related Sendai virus nucleocapsid peptide FAPGNYPAL and a very familiar human HLA (A*0201) related Influenza A virus matrix peptide GILGFVFTL have significantly lower Cα RMSD values of 0.25 Å and 0.16 Å respectively (Table 3) than those reported in earlier studies (2.70 Å and 0.46 Å, 1.60 Å, 1.40 Å respectively) [38,40-42] and those obtained using our previous protocol (0.40 Å and 0.32 Å). These observations establish the efficacy of pDOCK to dock highly accurate multi-species related peptide structures permitting conformational sampling of the peptide in the binding groove during flexible docking.

### Experiment 2

#### *Docking of multiple peptides onto a single template *

We applied pDOCK to a subset of 25 non-redundant pMHC complexes (obtained from the pDOCK test set of 186 pMHC complexes), with either a common allele or a common peptide core. The dataset of 18 MHC-I and seven MHC-II complexes comprises 21 (15 MHC-I and six MHC-II related) novel peptides which were known to bind to a single template (same allele) and four (three MHC-I and one MHC-II related) promiscuous peptides that were known to bind variant templates (multiple alleles). Due to lack of sufficient promiscuous peptides available in the PDB, only four peptides are currently tested. The results obtained from the docking of peptides onto single templates are tabulated in Table 4. 20 out of 21 peptides were docked onto a single template with Cα RMSD values less than 1.00 Å compared to their respective experimental structures. Amongst the results from single template docking experiments, the most accurate docked conformation of the least energy peptide, with a Cα RMSD of 0.06 Å compared to its relevant PDB peptide structure (Table 4), was achieved for the peptide from the structure 1kbg docked onto the MHC from the structure 1nam having the same murine MHC allele (H2-Kb) as the complex 1kbg.

**Table 4:** Docking novel peptides onto a single template: pDOCK compared to our previous method.

MHC class	Peptide PDB Allele	Peptide PDB	MHC Template Structure	Template Allele	Peptide Length	Peptide Sequence	Cα RMSD (Å)	
							
							Previous method	pDOCK
I	HLA-A*0201	2v2w	1qrn	HLA-A*0201	9	SLYNTVATL	0.63	0.38
I	HLA-A*0201	1hhh	1qrn	HLA-A*0201	10	F**LPSDFFPSV**	0.58	0.25
I	HLA-A*0201	1qse	1qrn	HLA-A*0201	9	LLFGYPRYV	0.62	0.30
I	HLA-A*0201	2bnq	1qrn	HLA-A*0201	9	SLLMWITQV	0.97	0.77
I	HLA-A*0201	2gj6	1qrn	HLA-A*0201	9	LLFGKPVYV	0.56	0.24
I	HLA-A*0201	1qr1	1qrn	HLA-A*0201	9	IISAVVGIL	0.87	0.36
I	HLA-A*0201	1qsf	1qrn	HLA-A*0201	9	LLFGYPVAV	0.94	0.41
I	HLA-A*0201	1bd2	1qrn	HLA-A*0201	9	LLFGYPVYV	0.68	0.46
I	HLA-A*0201	1hhg	1i4f	HLA-A*0201	9	TLTSCNTSV	0.58	0.56
I	HLA-A*0201	1hhh	1i4f	HLA-A*0201	10	F**LPSDFFPSV**	1.48	0.57
I	H2-Kb	1osz	1nam	H2-Kb	8	RGYLYQGL	0.85	0.47
I	H2-Kb	1fo0	1nam	H2-Kb	8	INFDFNTI	0.62	0.35
I	H2-Kb	1g6r	1nam	H2-Kb	8	SIYRYYGL	0.66	0.11
I	H2-Kb	1kbg	1nam	H2-Kb	8	RGYVYXGL	0.40	0.06
I	H2-Kb	1g7p	1nam	H2-Kb	9	SRDHSRTPM	1.41	0.82
II	HLA-DRB1*0101	1fyt	2iam	HLA-DRB1*0101	13	PK**YVKQNTLKL**AT	0.69	0.35
II	HLA-DRB1*0101	1klu	2iam	HLA-DRB1*0101	15	GEL**IGTLNAAKV**PAD	0.85	0.59
II	HLA-DRB1*0101	1t5w	2iam	HLA-DRB1*0101	13	AA**YSDQATPLL**LS	0.99	0.65
II	HLA-DRB1*0101	1pyw	2iam	HLA-DRB1*0101	9	FVKQNAXAL	0.40	0.32
II	HLA-DRB1*0101	1sje	2iam	HLA-DRB1*0101	15	PE**VIPMFSALS**EGAT	0.70	0.37
II	HLA-DRB1*0101	1aqd	2iam	HLA-DRB1*0101	14	GSD**WRFLRGYHQ**YA	1.68	1.01

Cα RMSD values are calculated only for the nonamer binding core (shown in bold) for peptides with more than 9 residues in the X-ray crystal structures.  X: Amino acid analogues (chemical mimics).

### Experiment 3

#### *Docking of same peptides onto variant templates *

Results from variant template docking experiments are listed in Table 5. It is worth noting that the Cα RMSD values for the peptides docked onto variant templates were calculated in comparison to the same peptides present in the respective template structures. This was done due to the fact that although the peptides may be similar, the environments encountered by the same peptides are different in the binding grooves of different MHC alleles. All four promiscuous peptides were docked onto variant templates with Cα RMSD values below 1.00 Å (Table 5). This observation suggests the robustness of pDOCK in docking promiscuous peptides onto multiple MHC alleles and its adaptability in ordering the binding registers or conformations of the peptides according to the changed environments, due to changes in the amino acid sequences, of the MHC grooves in different MHC alleles. Out of the 4 promiscuous peptides, the peptide FAPGNYPAL from the pMHC structure 2vaa having the murine MHC allele H2-Kb, when docked onto the MHC from the structure 1ce6 with the murine MHC allele H2-Db, was generated with the best Cα RMSD of 0.21 Å (Table 5) compared to the same peptide from 1ce6. The highest Cα RMSD value (0.79 Å) obtained using pDOCK during this experiment was when the peptide from the structure 1zsd was docked onto the MHC from the structure 2ak4 (Table 5). This value is still well within the acceptable value of 2.50 Å. 

**Table 5:** Docking promiscuous peptides onto variant templates: comparison of pDOCK with our previous method.

MHC class	Peptide PDB Allele	Peptide PDB	MHC Template Structure	Template Allele	Peptide Length	Peptide Sequence	Cα RMSD compared to template peptides (Å)	
							
							Previous method	pDOCK
I	HLA-B*3501	1zhk^#^	1zhl^#^	HLA-B*3508	13	LPEP**LPQGQLTAY**	0.62	0.44
I	HLA-B*3501	1zsd^ #^	2ak4	HLA-B*3508	11	EP**LPQGQLTAY**	1.15	0.79
I	H2-Kb	2vaa^#^	1ce6	H2-Db	9	FAPGNYPAL	0.73	0.21
II	HLA-DRB1*1501	1bx2^#^	1fv1	HLA-DRB5*0101	14	ENPV**VHFFKNIVT**P	1.01	0.22

Cα RMSD values are calculated only for the nonamer binding core (shown in bold) for peptides with more than 9 residues in the X-ray crystal structures. ^#^ The structures are not listed in Additional file – Table S1 due to redundancy in MPID-T2.

In all, only one peptide generated using pDOCK from the single template docking experiments has the Cα RMSD value above 1.00 Å (Table 4) compared to 5 peptides (three from single template docking and two from variant template docking) with Cα RMSD values above 1.00 Å using our previous methodology (Table 4 and Table 5). It is thus clear that pDOCK accurately predicts the structure of cognate peptides in both single and variant template docking cases. These evaluation steps are also vital to establish the efficiency with which our new method can dock and subsequently predict novel peptides onto given MHC proteins. 

## Conclusions

We have developed pDOCK as a fast, accurate and robust method for flexible docking of peptides to MHC-I and MHC-II proteins. Our results provide evidence of overcoming limitations pertaining to the application of our previous methodology, such as the presence of water molecules in and around the peptide and within the peptide binding groove in the template and relatively longer computational time required. Benchmarking with our previous method for a dataset of 50 non-redundant pMHC complexes consistently produced least energy docked conformations of peptides below 1.00 Å Cα RMSD from their respective native orientations for all 50 peptides. The Cα RMSD range for the same dataset was 0.09 Å (1s7q) to 0.66 Å (1i1y) using pDOCK compared to a Cα RMSD range from 0.20 Å (1s7q) to 1.53 Å (1jf1) applying our previous protocol. These observations imply an improvement in the accuracy by upto two and a half folds compared to our previous protocol. The outcomes of our validation experiments suggest a seven-fold improvement in the accuracy of the pDOCK docking protocol. pDOCK can therefore be successfully applied as a generalized, efficient protocol for docking of peptides to MHC-I and MHC-II receptors with improved accuracy, greater coverage of peptide residues and vastly reduced computational time (up to 60% compared to our earlier method). 

The average time taken to perform each step using pDOCK has also improved drastically compared to our old technique on a 2 CPU 3.20 GHz 3 GB RAM workstation. This is mainly due to the consolidation of the docking and refinement protocols into a single step docking and refinement procedure. Our results establish the efficacy of pDOCK to model highly accurate pMHC complex structures permitting conformational sampling of the peptide in MHC binding groove. The current study thus presents one of the most accurate pMHC docking protocols developed to date. pDOCK targets a more generic approach to generation of docked conformations of peptides using a single template for each allele. For some pMHC complexes however, appropriate addition of mediating molecules or considerations of solvent effects may lead to a possible improvement in docking accuracy. Rapid and large scale docking and scanning for identification of potential candidates for immunogenicity from repertoires of immunologically significant antigenic peptide sequences is possible by automating all the steps. No requirement for experimental data to be trained and the need of only a suitable template for a particular allele give pDOCK a prominent edge over other sequence-based techniques such as Artificial Neural Networks, Support Vector Machines, and Hidden Markov Models. 

pDOCK is also highly efficient in accurately predicting the docked conformations of amino acid analogues or chemical components within the peptide ligand suggesting its possible use as a docking and evaluation tool in structure-based drug design protocols and chemoinformatics. The single and variant template docking experiments along with the validation experiments also serve as strong benchmarks for pDOCK against our old method. pDOCK can correctly predict the conformation of residues that extend into the MHC binding cleft and therefore could help identify essential contacts with the MHC receptor, responsible for reducing the half life of the pMHC complex such that the peptide is held long enough within the MHC groove for presentation at the APC cell surface leading to surveillance and recognition by the TR molecules which in turn results in the activation of T cells and triggers the adaptive immune response cascade. Another significant improvement in this study is that the peptide ligand is allowed full flexibility within the peptide biding groove of the MHC proteins, unlike our previous method where the peptide termini were docked rigidly to the MHC groove. This aspect of pDOCK has helped us carry out fully flexible peptide docking to the MHC proteins. Our results also indicate the successful application of this protocol for easy *in silico* identification of promiscuous peptide epitopes that are applicable to higher proportions of human population with greater propensity to bind to MHC proteins and consequently activate T cells making them key targets for the design of vaccines and immunotherapies.

## Methods

### Data

pDOCK was tested on a non-redundant dataset of 186 (149 MHC-I and 37 MHC-II) pMHC complexes from the MPID-T2 (http://biolinfo.org/mpid-t2) database for which X-ray crystal structures are available in the PDB and the IMGT/3Dstructure-DB. When there is more than one complex with the same bound peptide and the same allele, the structure with the highest resolution is selected to avoid redundancy. When more than one bound peptide is available in the selected crystal structure, all bound peptides in that crystal structure are analyzed. TR/pMHC structures in MPID-T2 database are treated as non-redundant entries unless they have the same peptide, allele and TR type. In which case, the structure with the best resolution is considered non-redundant. Similarly, a dataset of 25 (18 MHC-I and 7 MHC-II) pMHC complexes was selected from the pDOCK test set for single and variant template docking. When more than one allele is available as template for docking of peptides into a single or variant template, the allele with the highest resolution was selected. Redundancy in MPID-T2 data is primarily decided from the similarities in peptides, MHC alleles and TR types (in case of TR/pMHC structures). Since one publication can refer to crystal structures of many complexes, redundancy in the literature is not considered as a criterion for redundancy. Some redundant structures were used for variant template docking (Table 5) due to limited number of crystal structures with promiscuous peptides bound to different alleles in the PDB. Although the MPID-T2 database contains 294 pMHC complexes (273 classical and 21 non classical), the 21 non-classical and 87 redundant structures were discarded from this study in order to avoid any biasness in our results.   

### pMHC complexes for benchmarking and validation

A non-redundant dataset of 50 high quality (35 MHC-I and 15 MHC-II) pMHC complexes, with maximum 3.00 Å resolutions, was selected from the 186 pMHC complexes in the pDOCK test set for benchmarking with the previous methodology. 15 pMHC complexes were chosen for validation experiments depending on the ones used in the corresponding reference studies [34,36-42]. 

### The pDOCK protocol

Unlike our earlier method [2,3], the new technique incorporates flexibility into the entire length of the peptide ligand. We have now incorporated a receptor modeling sub-step at the beginning of our novel schema (Figure 1), which involves rigorous homology modeling of MHC proteins from available MHC sequences by satisfaction of spatial restraints using MODELLER [43] followed by structure optimization and stringent structural quality assessment protocols to affirm the generation of high quality homology models of MHC proteins to be subsequently used in the pMHC docking strategy. Thereby, accounting for the validity of our methodology even in the absence of experimental structures for the MHC proteins and when only MHC sequences are available. However, this sub-step was not used in the current study as testing, benchmarking, validation, single template and variant template docking experiments are performed only on X-ray crystal structures of pMHC complexes.

The current pMHC docking technique is applied on MHC-I and MHC-II related peptides in two preparatory steps and a single consolidated docking and receptor step as follows: Preparatory step 1: receptor positioning using the Internal Coordinate Mechanics (ICM) global optimization algorithm [35]; Preparatory step 2: determining the docking grid using standardized values for MHC supertypes (MHC-I and MHC-II) from our preliminary studies and; A single docking and refinement step involving: ligand positioning, grid ligand docking followed by iterative ab initio refinements of backbone and ligand interacting side-chain dihedral angles of the MHC binding site residues to eliminate or minimize atomic clash regions at the pMHC interface using a Biased Monte Carlo procedure. The preparatory steps were together used to generate the receptor maps and the final single docking and refinement step was used to carry out ligand docking, generate the final least energy conformation and further refine the product. 

### Preparatory steps

#### *Receptor modeling and positioning*

Positioning of the MHC receptor is a major requirement in the pMHC docking simulation to ensure a best fit of the flexible peptide in the MHC groove. This first preparatory step (receptor modeling and positioning) is the least time consuming (only applicable to sub-step ‘b’) in the pDOCK docking protocol and involves two vital sub-steps: (a) homology model building by satisfaction of spatial restraints for MHC sequences where no structural data is available or inserting the MHC crystal structure into the docking simulation and; (b) positioning of the receptor within the docking simulation. Although not used in this study, high quality homology models can be generated, using our previously described three-step homology modeling procedure [44], for alleles with no structural data. Receptor positioning using the ICM global optimization algorithm assures the addition of any important missing residues in the template besides optimizing the zero occupancy side chains and any polar hydrogen atoms.

#### *Determining the docking grid*

**Figure 6:**
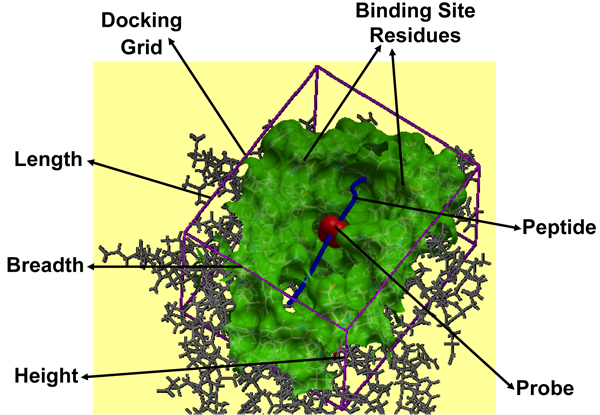
**pMHC docking caught in action.** Docking of the peptide into the MHC peptide binding ‘groove’ is shown for the pMHC complex 1zhb. The various components like binding site residues (green), docking grid (purple) dimensions (length, breadth and height), probe (red) and peptide (blue), involved in the peptide docking protocol are labeled.

The second, relatively small preparatory step of our docking procedure is to determining the docking grid which constitutes two major sub-steps: (a) defining the dimensions (*length* x *breadth* x *height*) of the 3-D docking grid and; (b) grid map generation for the receptor using the ICM stochastic global optimization algorithm. The ICM algorithm generates a three-dimensional docking grid (purple box in Figure 6), which encloses all MHC binding site residue atoms along with peptides residue atoms, soon after the previous step for generation of receptor maps. This ensures the localization of the peptide ligand for docking within the vicinity of the MHC peptide binding site residues and thereby limits the flexibility of the allowed peptide side chain torsion angles to be randomly sampled within the MHC groove. The dimensions of this 3-D docking box are set to standardized values derived from our preliminary analysis of all available pMHC complexes from the MPID-T2 database, for both MHC-I (35.36 Å x 35.52 Å x 35.79 Å) and MHC-II (58.32 Å x 56.36 Å x 48.87 Å) complexes used in testing, benchmarking, validation, single and variant template docking simulations. The ICM algorithm then selects all the binding site residues within the MHC groove by creating three-dimensional spheres from and around the centre of the MHC groove with 5.00 Å radii and selecting all the atoms of the MHC binding groove residues falling in and on the spheres (shown in green in Figure 6). 5.00 Å is set as the default radii to select the binding site residues amongst the residues forming the MHC groove as these are the MHC residues that are most likely to form hydrogen bonds (maximum allowed distance – 3.65 Å) and van der Waals contacts (maximum allowed distance – 4.50 Å) with the peptide residues, resulting in strong enough interactions to hold the bound peptide for presentation at the APC cell surface leading to surveillance and recognition by the TR molecules. The stochastic global optimization in internal coordinates with pseudo-Brownian and collective “probability-biased” random moves allow flexibility to the peptide ligand interface side chains and generate a grid potential map of the receptor energy localized to small cubic regions of side 1.00 Å from the carbon-alpha backbone of the peptide. 

### Single step docking and refinement

#### *Ligand positioning, grid ligand docking and refinement*

As with receptor positioning, ligand positioning is also equally important in achieving the best docked conformations, with the lowest energy values for flexible peptides using pDOCK. The final, most exhaustive (in terms of computational time required compared to the other two steps) single step docking and refinement part deals with ligand positioning, grid ligand docking and refinement, comprising three very important sub-steps: (a) positioning of the peptide ligand either by using the original crystal structure or by inserting a peptide model into the docking simulation using the peptide sequence; (b) placing and positioning of the probe into the peptide binding groove using the Internal Coordinate Mechanics global optimization algorithm and; (c) flexible docking of the peptide into the MHC groove and refinement of all ligand and binding site residues using a Biased Monte Carlo procedure. Ligand positioning was carried out either by using ICM algorithm for existing peptides within the X-ray crystal structures of pMHC complexes or by manually inserting a peptide model into the docking simulation for each of the available peptide sequences (docking of novel peptides to a single template and docking of promiscuous peptides to variant templates). This was followed by placing a probe (red in Figure 6) in the MHC groove which provides an initial position for conformational sampling and docking simulations using the ICM algorithm. 

ICM docking algorithm [35] runs flexible docking of peptide ligands to MHC peptide binding clefts. During the docking simulation, the ligand side-chain torsions that have been previously stored within the grid receptor maps (preparatory step 2) are changed in each random step using a Biased Monte Carlo procedure, which begins by pseudo-randomly selecting a set of torsion angles in the ligand and consequently finding the local energy minima about those angles. Upon satisfaction of the Metropolis criteria, novel conformations are adopted with a probability min (1, exp[−Δ*G/RT*]), where *R* is the universal gas constant and *T* is the absolute temperature of the simulation. The temperature was set to 300 K for the current study. To keep the ligand molecule close to the starting conformation, loose restraints are imposed on its positional variables. The internal energy function adopted for our simulations integrates internal van der Waals interactions energy (calculated using an extension of ECEPP/3 with force field parameters) [45], hydrogen bonding energy, torsion energy, electrostatic energy with a distance-dependent dielectric constant (ε = 4r; where ε is the distance-dependent dielectric constant and r is the distance) [46] and hydrophobic potential between the atoms of peptide residues and atoms of the binding site residues. The final energy incorporates configurational entropy of side chains and the surface-based solvation energy to select the best-iterated orientations. In brief, the complete optimal energy function, *E*,  is made up of the internal energy of the ligand and the intermolecular energy of the optimized receptor potential maps and can be summarized as: 

*E* = *E*_vw_  +   *E*_en_  +   2.16    +   2.53 *E*_hb_  +  4.35 *E*_hp_  +  0.20 *E*_solv_

where *E*_vw_ is the internal van der Waals interaction energy, *E*_en_ is the configurational/conformational entropy,    is the electrostatic energy of solvation, *E*_hb_ is the hydrogen bonding energy, *E*_hp_ is the hydrophobic potential and *E*_solv_ is the surface-based solvation energy. 

Finally, to improve the accuracy of the initial predicted conformation, refinement of the ligand as well as binding site residues backbone and side chains was performed as described in our previous methodology [2,3] to overcome any atomic clashes detected at the pMHC binding interface, using ICM Biased Monte Carlo procedure. Again, restraints are imposed upon the positional variables of the Cα atoms of the peptide residues. The early stages of the refinement efforts try to trounce the consequences of docking fully flexible ligands to rigid receptors by introducing partial flexibility to the backbone of MHC peptide binding residues. Refinements of ligand and receptor side-chain torsions in the vicinity of 4.00 Å from the receptor were executed upon the final backbone structure of the peptides to keep the docked peptides closest to their starting conformations. The energy function, *E*, utilized for this refinement sub-step, is the sum of energy terms arising from the optimal energy function described above:

*E* = *E*_vw_  +  *E*_hbonds_  +  *E*_tors_  +  *E*_elec_  +  *E*_solv_  +  *E*_en_

where *E*_tors_ is the torsion energy, *E*_elec_ is the electrostatic energy and *E*_en_ is the entropic term.

## Competing interests

The authors declare that they have no competing interests.

## Authors’ contributions

JMK developed the methodology, carried out the computational simulation studies and drafted the manuscript. JMK and SR participated in the design of the study and interpretation of data. SR conceived the project and finalized the manuscript. Both authors have read and approved the final manuscript.

## Supplementary Material

Additional File 1 Application of pDOCK to the 186 (149 MHC-I and 37 MHC-II) non-redundant structures from MPID-T2 database. Click here for file

Additional File 2 Comparison of Cα RMSD values obtained using pDOCK and our previous method across the benchmarking dataset Click here for file
