# Supplementary material for: pDOCK: a new technique for rapid and accurate docking of peptide ligands to Major Histocompatibility Complexes
Source: Immunome Res. 2010 Sep 27;6(Suppl 1):S2. doi: 10.1186/1745-7580-6-S1-S2 (PMC2946780; doi:10.1186/1745-7580-6-S1-S2)
Supplement: Additional File 1 — Application of pDOCK to the 186 (149 MHC-I and 37 MHC-II) non-redundant structures from MPID-T2 database. [file 1745-7580-6-S1-S2-S1.pdf]

## Additional File 1

**pDOCK: A new technique for rapid and accurate docking of peptide ligands to Major Histocompatibility Complexes**

Javed M. Khan and Shoba Ranganathan

**Table S1 – Application of pDOCK to the 186 (149 MHC-I and 37 MHC-II) non-redundant structures from MPID-T2 database.**

C$\alpha$ RMSD values are calculated only for the nonameric core (shown in bold) forming the MHC binding register for peptides with more than 9 residues in the X-ray crystal structures. X: Chemical mimics.

| Allele | PDB code | Peptide Sequence | Peptide Length | Res. (Å) | Cα RMSD (Å) |
|---|---|---|---|---|---|
| **MHC-I** | | | | | |
| HLA-A*0101 | 1w72 | EADPTGHSY | 9 | 2.15 | 0.42 |
| HLA-A*1101 | 1qvo | Q**VPLRPMTYK** | 10 | 2.22 | 0.24 |
| HLA-A*1101 | 1x7q | KTFPPTEPK | 9 | 1.45 | 0.36 |
| HLA-A*1101 | 2hn7 | A**IMPARFYPK** | 10 | 1.60 | 1.40 |
| HLA-A*1101 | 1q94 | AIFQSSMTK | 9 | 2.40 | 1.52 |
| HLA-A*0201 | 1oga | GILGFVFTL | 9 | 1.40 | 0.16 |
| HLA-A*0201 | 1t1y | SLYNVVATL | 9 | 2.00 | 0.78 |
| HLA-A*0201 | 1s8d | SLANTVATL | 9 | 2.20 | 0.65 |
| HLA-A*0201 | 2gtz | ALGIGILTV | 9 | 1.70 | 0.95 |
| HLA-A*0201 | 1duy | LFGYPVYV | 8 | 2.15 | 1.12 |
| HLA-A*0201 | 2p5w | SLLMWITQC | 9 | 2.20 | 0.35 |
| HLA-A*0201 | 2v2w | SLYNTVATL | 9 | 1.60 | 0.55 |
| HLA-A*0201 | 2v2x | SLFNTVATL | 9 | 1.60 | 0.78 |
| HLA-A*0201 | 1qr1 | IISAVVGIL | 9 | 2.40 | 0.29 |
| HLA-A*0201 | 1qrn | LLFGYAVYV | 9 | 2.80 | 0.91 |
| HLA-A*0201 | 1qse | LLFGYPRYV | 9 | 2.80 | 0.31 |
| HLA-A*0201 | 1qsf | LLFGYPVAV | 9 | 2.80 | 0.34 |
| HLA-A*0201 | 1hhg | TLTSCNTSV | 9 | 2.60 | 1.18 |
| HLA-A*0201 | 1lp9 | ALWGFFPVL | 9 | 2.00 | 0.26 |
| HLA-A*0201 | 1s9x | SLLMWITQA | 9 | 2.50 | 0.44 |
| HLA-A*0201 | 1s9y | SLLMWITQS | 9 | 2.30 | 0.39 |
| HLA-A*0201 | 1tvb | ITDQVPFSV | 9 | 1.80 | 0.38 |
| HLA-A*0201 | 1tvh | IMDQVPFSV | 9 | 1.80 | 0.43 |
| HLA-A*0201 | 1akj | ILKEPVHGV | 9 | 2.65 | 0.39 |
| HLA-A*0201 | 1eey | ILSALVGIV | 9 | 2.25 | 0.92 |
| HLA-A*0201 | 2guo | AAGIGILTV | 9 | 1.90 | 2.14 |
| HLA-A*0201 | 1t20 | SLYNTIATL | 9 | 2.20 | 0.82 |
| HLA-A*0201 | 1ao7 | LLFGYPVYV | 9 | 2.60 | 0.58 |
| HLA-A*0201 | 1eez | ILSALVGIL | 9 | 2.30 | 0.71 |

| Allele | PDB code | Peptide Sequence | Peptide Length | Res. (Å) | Cα RMSD (Å) |
|---|---|---|---|---|---|
| HLA-A*0201 | 2gj6 | LLFGKPVYV | 9 | 2.56 | 0.97 |
| HLA-A*0201 | 1hhh | F**LPSDFFPSV** | 10 | 3.00 | 0.49 |
| HLA-A*0201 | 1i1f | FLKEPVHGV | 9 | 2.80 | 0.64 |
| HLA-A*0201 | 2clr | **MLLSVPLLI**G | 10 | 2.00 | 1.01 |
| HLA-A*0201 | 2gt9 | E**AAGIGILTV** | 10 | 1.75 | 0.26 |
| HLA-A*0201 | 1i7r | FAPGFFPYL | 9 | 2.20 | 0.47 |
| HLA-A*0201 | 1i7t | ALWGVFPVL | 9 | 2.80 | 0.42 |
| HLA-A*0201 | 1i7u | ALWGFVPVL | 9 | 1.80 | 0.29 |
| HLA-A*0201 | 1jf1 | E**LAGIGILTV** | 10 | 1.85 | 0.30 |
| HLA-A*0201 | 1t1x | SLYLTVATL | 9 | 2.20 | 1.72 |
| HLA-A*0201 | 1i1y | YLKEPVHGV | 9 | 2.20 | 0.66 |
| HLA-A*0201 | 1t1z | ALYNTAAAL | 9 | 1.90 | 1.15 |
| HLA-A*0201 | 2bnq | SLLMWITQV | 9 | 1.70 | 0.55 |
| HLA-A*0201 | 2c7u | SLFNTIAVL | 9 | 2.38 | 0.86 |
| HLA-A*0201 | 1i4f | G**VYDGREHTV** | 10 | 1.40 | 1.25 |
| HLA-A*0201 | 1bd2 | LLFGYPVYV | 9 | 2.50 | 0.33 |
| HLA-A*0201 | 2gtw | LAGIGILTV | 9 | 1.55 | 3.08 |
| HLA-A*2402 | 2bck | VYGFVRACL | 9 | 2.80 | 0.79 |
| HLA-A*6801 | 1tmc | E**VAPPEYHRK** | 10 | 2.30 | 0.54 |
| HLA-B*0801 | 1agc | GGKKKYQL | 8 | 2.10 | 0.23 |
| HLA-B*0801 | 1agd | GGKKKYKL | 8 | 2.05 | 0.45 |
| HLA-B*0801 | 1agb | GGRKKYKL | 8 | 2.20 | 0.28 |
| HLA-B*0801 | 1mi5 | FLRGRAYGL | 9 | 2.50 | 0.37 |
| HLA-B*1501 | 1xr8 | LEKARGSTY | 9 | 2.30 | 1.15 |
| HLA-B*1501 | 3c9n | VQQESSFVM | 9 | 1.87 | 0.80 |
| HLA-B*2101 | 3bev | GH**AEEYGAETL** | 11 | 2.10 | 0.90 |
| HLA-B*2101 | 3bew | R**EVDEQLLSV** | 10 | 2.60 | 0.33 |
| HLA-B*2705 | 1uxs | RRRWRRLTV | 9 | 1.55 | 0.75 |
| HLA-B*2705 | 1ogt | RRKWRRWHL | 9 | 1.47 | 0.18 |
| HLA-B*2705 | 2bsr | RRIYDLIEL | 9 | 2.30 | 1.72 |
| HLA-B*2705 | 2bst | SRYWAIRTR | 9 | 2.10 | 1.36 |
| HLA-B*2705 | 2a83 | RRRWHRWRL | 9 | 1.40 | 0.18 |
| HLA-B*2705 | 1w0v | RRLPIFSRL | 9 | 2.27 | 1.41 |
| HLA-B*2709 | 1w0w | RRLPIFSRL | 9 | 2.10 | 0.64 |
| HLA-B*2709 | 1k5n | GRFAAAIAK | 9 | 1.09 | 0.75 |
| HLA-B*2709 | 1uxw | RRRWRRLTV | 9 | 1.71 | 0.47 |
| HLA-B*2709 | 1jgd | R**RLLRGHNQY** | 10 | 1.90 | 1.42 |
| HLA-B*3501 | 2cik | KPIVVLHGY | 9 | 1.75 | 0.26 |
| HLA-B*3501 | 2axg | A**PQPAPENAY** | 10 | 2.00 | 1.14 |
| HLA-B*3501 | 1a9b | LPPLDITPY | 9 | 3.20 | 0.39 |
| HLA-B*3501 | 1qew | FLWGPRALV | 9 | 2.20 | 0.53 |
| HLA-B*3501 | 1a1n | VPLRPMTY | 8 | 2.00 | 0.39 |
| HLA-B*3508 | 3bw9 | CPS**QEPMSIYVY** | 12 | 1.75 | 0.27 |
| HLA-B*3508 | 3bwA | FPTKDVAL | 8 | 1.30 | 0.26 |
| HLA-B*3508 | 2ak4 | LPEP**LPQGQLTAY** | 13 | 2.50 | 1.09 |

| Allele | PDB code | Peptide Sequence | Peptide Length | Res. (Å) | Cα RMSD (Å) |
|---|---|---|---|---|---|
| HLA-B*3508 | 2axf | A**PQPAPENAY** | 10 | 1.80 | 0.51 |
| HLA-B*4402 | 1m6o | EEFGRAFSF | 9 | 1.60 | 1.33 |
| HLA-B*4403 | 1sys | EEPTVIKKY | 9 | 2.40 | 0.65 |
| HLA-B*4403 | 1n2r | EEFGRAFSF | 9 | 1.70 | 0.99 |
| HLA-B*4405 | 1syv | EEFGRAFSF | 9 | 1.70 | 0.77 |
| HLA-B*5101 | 1e27 | LPPVVAKEI | 9 | 2.20 | 0.18 |
| HLA-B*5101 | 1e28 | TAFTIPSI | 8 | 3.00 | 0.26 |
| HLA-B*5301 | 1a1m | TPYDINQML | 9 | 2.30 | 0.28 |
| HLA-B*5301 | 1a1o | KPIVQYDNF | 9 | 2.30 | 0.84 |
| HLA-B*5703 | 2bvq | KAFSPEVIP | 9 | 2.00 | 0.50 |
| HLA-B*5703 | 2bvo | KA**FSPEVIPMF** | 11 | 1.65 | 2.17 |
| HLA-B*5703 | 2bvp | ISPRTLDAW | 9 | 1.35 | 0.65 |
| HLA-Cw*0304 | 1efx | GAVDPLLAL | 9 | 3.00 | 0.35 |
| HLA-Cw*0401 | 1im9 | QYDDAVYKL | 9 | 2.80 | 0.34 |
| HLA-E*0101 | 2esv | VMAPRTLIL | 9 | 2.60 | 0.66 |
| HLA-E*0103 | 1kpr | VMAPRTVLL | 9 | 2.80 | 1.02 |
| HLA-E*0103 | 1ktl | VTAPRTLLL | 9 | 3.10 | 0.45 |
| HLA-E*0103 | 3cdg | VMAPRTLFL | 9 | 3.40 | 1.97 |
| HLA-G*0101 | 2dyp | RIIPRHLQL | 9 | 2.50 | 0.16 |
| H2-Db | 1jpf | SG**VENPGGYCL** | 11 | 2.18 | 0.36 |
| H2-Db | 1jpg | FQPQNGQFI | 9 | 2.20 | 0.38 |
| H2-Db | 1juf | SSVIGVWYL | 9 | 2.00 | 0.29 |
| H2-Db | 1bz9 | FAPGVFPYM | 9 | 2.80 | 1.19 |
| H2-Db | 1ce6 | FAPGNYPAL | 9 | 2.90 | 0.24 |
| H2-Db | 1hoc | ASNENMETM | 9 | 2.40 | 0.46 |
| H2-Db | 1ffo | AAVYNFATM | 9 | 2.65 | 0.25 |
| H2-Db | 1ffp | SAVYNFATM | 9 | 2.60 | 0.33 |
| H2-Db | 1fg2 | KAVYNFATC | 9 | 2.75 | 0.19 |
| H2-Db | 1inq | SSVVGVWYL | 9 | 2.20 | 0.42 |
| H2-Db | 1n3n | S**NLQNAASIA** | 10 | 3.00 | 1.34 |
| H2-Db | 1qlf | FAPSNYPAL | 9 | 2.65 | 0.26 |
| H2-Db | 1s7v | KAVYNLATM | 9 | 2.20 | 0.27 |
| H2-Db | 1s7w | KALYNFATM | 9 | 2.40 | 0.62 |
| H2-Db | 1s7x | KAVFNFATM | 9 | 2.41 | 0.47 |
| H2-Db | 1wbx | S**QLKNNAKEI** | 10 | 1.90 | 0.38 |
| H2-Db | 1wby | S**SLENFRAYV** | 10 | 2.30 | 0.31 |
| H2-Db | 1yn7 | S**SLENFAAYV** | 10 | 2.20 | 0.14 |
| H2-Db | 2f74 | KAVYNFATM | 9 | 2.70 | 0.27 |
| H2-Db | 3buy | LSLRNPILV | 9 | 2.60 | 0.23 |
| H2-Dd | 1qo3 | R**GPGRAFVTI** | 10 | 2.30 | 0.17 |
| H2-Kb | 1g6r | SIYRYYGL | 8 | 2.80 | 0.34 |
| H2-Kb | 1s7q | KAVYNFATM | 9 | 1.99 | 0.09 |
| H2-Kb | 1s7r | KAVYNLATM | 9 | 2.95 | 1.26 |
| H2-Kb | 1s7s | KALYNFATM | 9 | 1.99 | 0.28 |
| H2-Kb | 1s7t | KAVFNFATM | 9 | 2.30 | 0.19 |

| Allele | PDB code | Peptide Sequence | Peptide Length | Res. (Å) | Cα RMSD (Å) |
|---|---|---|---|---|---|
| H2-Kb | 1g7p | SRDHSRTPM | 9 | 1.50 | 0.17 |
| H2-Kb | 1g7q | SAPDTRPA | 8 | 1.60 | 0.36 |
| H2-Kb | 1kbg | RGYVYXGL | 8 | 2.20 | 0.47 |
| H2-Kb | 1t0m | SSIEFARL | 8 | 2.00 | 0.21 |
| H2-Kb | 1vac | SIINFEKL | 8 | 2.50 | 0.22 |
| H2-Kb | 1wbz | SSYRRPVGI | 9 | 2.00 | 0.19 |
| H2-Kb | 1rjz | SEIEFARL | 8 | 2.60 | 0.48 |
| H2-Kb | 1kj2 | KVITFIDL | 8 | 2.71 | 0.38 |
| H2-Kb | 1lk2 | GNYSFYAL | 8 | 1.35 | 0.53 |
| H2-Kb | 1zhb | KALYNYAPI | 9 | 2.70 | 0.24 |
| H2-Kb | 1mwa | EQYKFYSV | 8 | 2.40 | 0.27 |
| H2-Kb | 2fo4 | SAPDFRPL | 8 | 2.70 | 0.60 |
| H2-Kb | 1n59 | AVYNFATM | 8 | 2.95 | 0.44 |
| H2-Kb | 2ol3 | SQYYYNSL | 8 | 2.90 | 0.30 |
| H2-Kb | 1nam | RGYVYQGL | 8 | 2.70 | 0.38 |
| H2-Kb | 1fo0 | INFDFNTI | 8 | 2.50 | 0.34 |
| H2-Kb | 1osz | RGYLYQGL | 8 | 2.10 | 0.28 |
| H2-Kd | 1vgk | SYVNTNMGL | 9 | 2.06 | 0.25 |
| H2-Kd | 2fwo | TYQRTRALV | 9 | 2.60 | 0.26 |
| H2-Kk | 1zt1 | FEANGNLI | 8 | 2.50 | 0.45 |
| H2-Kk | 1zt7 | SEFLLEKRI | 9 | 3.00 | 0.45 |
| H2-Ld | 1ldp | APAAAAAAM | 9 | 3.10 | 0.59 |
| H2-Ld | 1ld9 | YPNVNIHNF | 9 | 2.40 | 0.56 |
| H2-Ld | 2e7l | QLSPFPFDL | 9 | 2.50 | 0.35 |
| H2-Ld | 2oi9 | QLSPFPFDL | 9 | 2.35 | 0.55 |
| H2-M3 | 1mhc | MYFINILTL | 9 | 2.10 | 1.16 |
| H2-Qa-2 | 1k8d | ILMEHIHKL | 9 | 2.30 | 0.55 |
| Mamu-A*01 | 1zvs | TTPESANL | 8 | 2.80 | 0.65 |
| RT1.Aa | 1kjm | AQFSASASR | 9 | 2.35 | 0.49 |
| RT1-A1C | 1kjv | NPRAMQALL | 9 | 1.48 | 0.33 |
| **MHC-II** | | | | | |
| HLA-DQB1*0201 | 1s9v | LQ**PFPQPELPY** | 11 | 2.22 | 0.33 |
| HLA-DQB1*0302 | 1jk8 | LV**EALYLVCGE**RGG | 14 | 2.40 | 0.31 |
| HLA-DQB1*0302 | 2nna | SG**EGSFQPSQE**NP | 13 | 2.10 | 0.22 |
| HLA-DQB1*0602 | 1uvq | MN**LPSTKVSWA**AVGGGGSLV | 20 | 1.80 | 0.23 |
| HLA-DRA*0101 | 1zgl | VHH**FKNIVTPRT**PG | 14 | 2.80 | 1.27 |
| HLA-DRB1*0101 | 1aqd | GSD**WRFLRGYHQ**YA | 14 | 2.45 | 0.28 |
| HLA-DRB1*0101 | 1fyt | PK**YVKQNTLKL**AT | 13 | 2.60 | 0.23 |
| HLA-DRB1*0101 | 1klu | GEL**IGTLNAAKV**PAD | 15 | 1.93 | 0.20 |
| HLA-DRB1*0101 | 1pyw | FVKQNAXAL | 9 | 2.10 | 0.81 |
| HLA-DRB1*0101 | 1sje | PE**VIPMFSALS**EGAT | 15 | 2.45 | 0.46 |
| HLA-DRB1*0101 | 1sjh | PE**VIPMFSALS**EG | 13 | 2.25 | 0.22 |
| HLA-DRB1*0101 | 1t5w | AA**YSDQATPLL**LS | 13 | 2.40 | 0.25 |
| HLA-DRB1*0101 | 2fse | AG**FKGEQGPKG**EPG | 14 | 3.10 | 0.64 |

| Allele | PDB code | Peptide Sequence | Peptide Length | Res. (Å) | Cα RMSD (Å) |
|---|---|---|---|---|---|
| HLA-DRB1*0101 | 2iam | GEL**IGILNAAKV**PAD | 15 | 2.80 | 0.24 |
| HLA-DRB1*0301 | 1a6a | PVSK**MRMATPLLM**QA | 15 | 2.75 | 0.30 |
| HLA-DRB1*0401 | 1d5m | XXRAMXSX | 8 | 2.00 | 0.13 |
| HLA-DRB1*0401 | 1d5x | XXRXXX | 6 | 2.45 | 0.11 |
| HLA-DRB1*0401 | 1d5z | XXRAXSX | 7 | 2.00 | 0.22 |
| HLA-DRB1*0401 | 1d6e | XXRXMASX | 8 | 2.45 | 0.14 |
| HLA-DRB1*0401 | 1j8h | PK**YVKQNTLKL**AT | 13 | 2.40 | 0.20 |
| HLA-DRB1*0401 | 2seb | AY**MRADAAAGG**A | 12 | 2.50 | 0.31 |
| HLA-DRB1*1501 | 1ymm | ENPV**VHFFKNIVT**P | 14 | 3.50 | 0.28 |
| HLA-DRB3*0101 | 2q6w | A**WRSDEALPL**G | 11 | 2.25 | 0.30 |
| HLA-DRB5*0101 | 1fv1 | NPVVHF**FKNIVTPRT**PPPSQ | 20 | 1.90 | 0.59 |
| HLA-DRB5*0101 | 1h15 | GGV**YHFVKKHVH**ES | 14 | 3.10 | 0.22 |
| HLA-DRB5*0101 | 1hqr | V**HFFKNIVTP** | 10 | 3.20 | 0.56 |
| I-Ab | 1muj | PVSK**MRMATPLLM**QA | 15 | 2.15 | 0.15 |
| I-Ad | 1iao | RGI**SQAVHAAHA**EI | 14 | 2.60 | 0.27 |
| I-Ad | 2iad | GHA**TQGVTAASS**HE | 14 | 2.40 | 0.56 |
| I-A(G7) | 1es0 | YE**IAPVFVLLE**YVT | 14 | 2.60 | 0.38 |
| I-Ak | 1f3j | AMK**RHGLDNYRG**YS | 14 | 3.10 | 0.28 |
| I-Ak | 1iak | ST**DYGILQINS**RW | 13 | 1.90 | 0.23 |
| I-Ak | 1jl4 | GNS**HRGAIEWEG**IESG | 16 | 4.30 | 0.35 |
| I-Au | 1u3h | SR**GGASQYRPS**Q | 12 | 2.42 | 0.95 |
| I-Au | 2pxy | R**GGASQYRPS**Q | 11 | 2.23 | 0.28 |
| I-Ek | 1r5v | ADL**IAYPKAATK**F | 13 | 2.50 | 0.28 |
| I-Ek | 1r5w | ADL**IAYFKAATK**F | 13 | 2.90 | 1.26 |
