# Supplementary material for: pDOCK: a new technique for rapid and accurate docking of peptide ligands to Major Histocompatibility Complexes
Source: Immunome Res. 2010 Sep 27;6(Suppl 1):S2. doi: 10.1186/1745-7580-6-S1-S2 (PMC2946780; doi:10.1186/1745-7580-6-S1-S2)

Additional File 2

pDOCK: A new technique for rapid and accurate docking of peptide ligands to Major Histocompatibility Complexes

Javed M. Khan and Shoba Ranganathan

Figure S1 – Comparison of Cα RMSD values obtained using pDOCK and our previous method across the benchmarking dataset.

The pMHC complex number refers to the serial number of the complexes listed in Table 1. The most significant pDOCK results are highlighted in yellow.

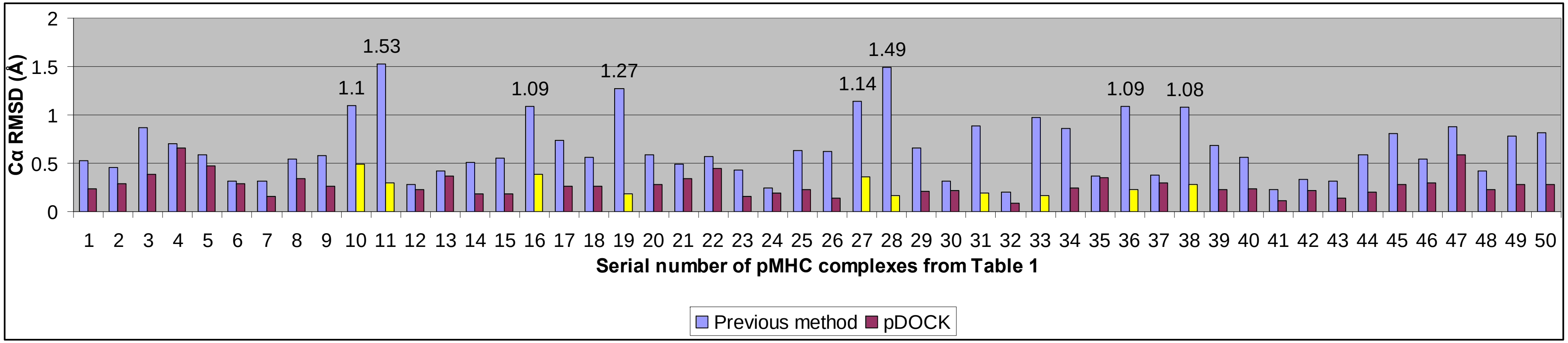

Supplement: Additional File 2 — Comparison of Cα RMSD values obtained using pDOCK and our previous method across the benchmarking dataset [file 1745-7580-6-S1-S2-S2.pdf]
